# English and Foreign Hospitals

**Published:** 1899-11-25

**Authors:** 


### ENGLISH AND FOREIGN HOSPITALS.

The following interesting comment on the characteristics of English hospitals appears in the *Medical News* of New York: When the teaching University of London adds its powerful impulse to the others already in operation there can be little question that a great centralised scheme of post-graduate teaching and probably a great central teaching institution will grow up which will put London upon the same plane with Vienna, Berlin, and Paris as a medical Mecca. One great obstacle to this scheme of centralising at least the clinical part of medical teaching exists in the nature of the situation, and is not always, perhaps, appreciated at its full value. This is the intensely individualistic character which pervades not only all English institutions but all classes of English society, and is strikingly illustrated in the fact of there being eight self-supporting and totally independent medical schools instead of one central State-supported and regulated University, as in Berlin or Leipzig, and there will naturally be a distinct amount of friction before these can be got to fuse their differences in a common scheme. On the other hand, the patient himself is unfortunately more individualistic, in the widest and most aggressive sense, than his French or German brother. He will go to the hospital that he chooses in the first place and distinctly refuse to budge to another no matter what inducements may be offered him in the way of superior advice, and he will object to being seen by strange doctors and strange students. He will absolutely refuse to be operated upon unless the procedure commends itself to his individual judgment; his relatives after his death will bitterly oppose the use of his remains for post-mortem study, and in a score of other ways he is often a serious practical obstacle in the way of systematic clinical work. Nor does he stand alone in this, for it may never be forgotten that all English hospitals are organised for the benefit of their patients first and foremost, and it is often with the greatest difficulty that even the staff of the hospital can get permission to use its inmates for clinical purposes within its own walls. Any complaint of unnecessarily frequent or prolonged examination on the part of the patient, or of fatigue in being transported to another lecture-room, or even of outraged modesty from being submitted to the gaze of a large audience of students, will be promptly taken up and supported, not merely by the board of governors of the hospital, but also by the outside public, upon whose voluntary contributions the institution in most cases absolutely depends. However, and happily, both patients, governors, and the laity are coming to recognise the true value and advantage of expert opinion and additional advice. The more intelligent class of patients is coming to respond readily to any suggestions as to methods of obtaining more light upon their cases. The governors are finding that the hospital that does the best and most effective therapeutic work, and the whole feeling of even the more ignorant class of the laity toward the profession is improving most pleasantly. There can be no question that both this sort of feeling on the patient's part, and its frank and manly recognition on the part of the operator and consultant, has a most valuable influence in keeping English and American hospital practice upon the highest plane of humanity, of courtesy, and in the broadest sense of true science, than that which obtains in some Continental centres.

